# Membrane-Initiated Non-Genomic Signaling by Estrogens in the Hypothalamus: Cross-Talk with Glucocorticoids with Implications for Behavior

**DOI:** 10.3389/fendo.2015.00018

**Published:** 2015-02-16

**Authors:** Jennifer Rainville, Kevin Pollard, Nandini Vasudevan

**Affiliations:** ^1^Department of Cell and Molecular Biology, Tulane University, New Orleans, LA, USA; ^2^Neuroscience Program, Tulane University, New Orleans, LA, USA

**Keywords:** hypothalamus, spine density, membrane-initiated signaling, GPCR, estrogen receptor variants, aggression, lordosis, glucocorticoid receptor

## Abstract

The estrogen receptor and glucocorticoid receptor are members of the nuclear receptor superfamily that can signal using both non-genomic and genomic transcriptional modes. Though genomic modes of signaling have been well characterized and several behaviors attributed to this signaling mechanism, the physiological significance of non-genomic modes of signaling has not been well understood. This has partly been due to the controversy regarding the identity of the membrane ER (mER) or membrane GR (mGR) that may mediate rapid, non-genomic signaling and the downstream signaling cascades that may result as a consequence of steroid ligands binding the mER or the mGR. Both estrogens and glucocorticoids exert a number of actions on the hypothalamus, including feedback. This review focuses on the various candidates for the mER or mGR in the hypothalamus and the contribution of non-genomic signaling to classical hypothalamically driven behaviors and changes in neuronal morphology. It also attempts to categorize some of the possible functions of non-genomic signaling at both the cellular level and at the organismal level that are relevant for behavior, including some behaviors that are regulated by both estrogens and glucocorticoids in a potentially synergistic manner. Lastly, it attempts to show that steroid signaling via non-genomic modes may provide the organism with rapid behavioral responses to stimuli.

## Genomic and Non-Genomic Signaling by Nuclear Receptors

Nuclear receptor ligands such as estrogen and glucocorticoids signal via both non-genomic and genomic pathways within cells. The genomic or transcriptional pathway is the best elucidated primarily due to the well-characterized nature of the estrogen receptor (ER)α and β and the glucocorticoid receptor (GR), all of which are members of the nuclear receptor superfamily. Once bound to their cognate ligands, these receptors act as ligand-activated transcription factors in the nucleus by binding to specific enhancer elements such as the estrogen response element (ERE) (1) and glucocorticoid response element (GRE) (2) in the promoters of genes. Both receptors have a modular structure, with a conserved DNA-binding domain, multiple transactivation domains, and a C-terminal ligand-binding domain (3, 4).

On the other hand, non-genomic signaling, first described by Szego and Davis in 1967, as the rapid increase in cAMP in the uterus occurred within 15 min of 17β-estradiol (17β-E) administration to ovariectomized mice (5). In the central nervous system (CNS), 17β-E was shown to rapidly depolarize pro-opiomelanocortin (POMC) hypothalamic neurons via Akt or protein kinase (PK) B, extracellular regulated kinase (ERK/MAPK), PKA, and PKC pathways (6, 7). In other tissues such as rat hippocampal neurons, phospho-cAMP response element binding protein (pCREB) increased within 1 h of 17β-E addition and this increase was blocked by inhibitors to both calmodulin kinase II (CamKII) and ERK pathways (8). In the case of corticosterone-mediated rapid actions, treatment of neurons with dexamethasone, a synthetic glucocorticoid, rapidly induced the nuclear localization of the GR (9, 10), an effect potentiated by the inhibition of p38MAPK (11). Extracts from rat hippocampal synaptoneurosomes showed a reduction in Akt and ERK phosphorylation within 30 min in response to pharmacological inhibition of the GR by RU-486 (12), suggesting that the classical nuclear receptor was required for non-genomic signaling in the hippocampus. Apart from kinase activation, dexamethasone-mediated negative feedback at the corticotropin releasing hormone (CRH) neuron was also rapid, consisting of suppression of the excitatory drive to the CRH neuron, mediated by endocannabinoids acting as a retrograde messenger to the presynaptic glutamatergic neuron (13), an effect mimicked with a membrane-limited dexamethasone conjugated to bovine serum albumin (Dex-BSA) (13). Hence, non-genomic signaling by steroid hormones is extra-nuclear signaling that is initiated by the endogenous ligand within minutes, in contrast to the hours required to detect transcriptionally regulated proteins.

Central to this concept of non-genomic signaling that is typically demonstrated by the use of membrane-limited conjugates (14), is the idea of a receptor that initiates such signaling from the plasma membrane. However, with the exception of the membrane progesterone receptors (mPRs) that belong to the progestin and adipoQ receptor (PAQR) family, the identity of the membrane ER (mER) and membrane GR (mGR) has remained elusive (15). This review aims to describe the current candidates for the mER and the mGR that mediate rapid non-genomic signaling from the plasma cell membrane as well as focus on rapid actions that are relevant for hypothalamically driven behaviors that are dependent on estrogens but that have a glucocorticoid-regulated component. We concentrate on the hypothalamus since this is a classically steroid-responsive area of the brain and is critical for several estrogen-dependent behaviors (16). For more general reviews on rapid actions of estrogens and glucocorticoids in the CNS, including tools that are typically used to elucidate membrane-initiated non-genomic effects, the reader is referred to (7, 14, 17–19).

## The Membrane ER and GR

In the CNS, both ^3^H-labeled 17β-E (20) and a ^125^I-labeled membrane-impermeant conjugate where 17β-E is attached to bovine serum albumin (E2-BSA) (21) showed relatively high affinity binding to rat plasma membranes, suggesting the presence of a mER. What is a good definition for a mER or mGR? Previously, Micevych et al. (22) have suggested that ICI 182,780 antagonism, stereospecific 17β-E binding and sequence homology to the ERα and ERβ should be considered pre-requisites for a protein to be termed a mER. We propose, given the off-target effects of ICI 182,780 (23) that the definition of the mER or mGR be modified slightly to consider proteins capable of specific binding to 17β-E or to dexamethasone (in the case of the mGR), presence at/near the plasma membrane and signaling from the membrane. It should be noted that this definition would exclude the binding of proteins to 17α-E, an estrogen often used in studies of as an inactive isomer of 17β-estradiol, but that recently has been shown to increase neurogenesis and to mediate neuroprotection (24). Most studies described below show evidence of the candidate mER at the plasma membrane and its ability to rapidly within minutes modulate rapid signaling pathways such as kinase regulation or calcium flux. A few studies also demonstrate the regulation of the candidate ER at the membrane.

### ERα as the mER

As we will focus on outputs dependent on the hypothalamus in this review, we will initially discuss the nuclear classical ERα as a candidate mER because (a) this is the most abundant ER isoform in the medial preoptic area (mPOA), ventromedial hypothalamus (VMH), and arcuate nucleus (ARH) (25–27) and (b) loss of ERα in the hypothalamus abrogated hypothalamically driven lordosis behavior in females (28) and aggressive behavior in males (29). A number of studies using 17β-E binding, electron microscopy, immunocytochemistry, and western blotting have examined the idea that a small percentage (3–5%) of the total pool of the classical ERα is present on the plasma membrane (30). Consistent with this idea, immunocytochemistry using minimal fixation in both the breast cancer MCF-7 cell line (31) and anterior pituitary GH3 cell line (32), revealed ERα at the membrane in caveolae in some, but not all cells. MCF-7 cells with ERα at the plasma membrane showed increased phospho-ERK (pERK) within 10 min of application of either 17β-E or a membrane-limited E2-peroxidase conjugate (33). In the CNS, ERα was localized to the dendrites and axon terminals in the guinea pig hypothalamus (34), while ERα was detected in axon terminals, dendritic spines, as well as in astrocytes in the CA1 using immuno-electron microscopy in the proestrous female rat (35). In the dorsal CA1 from the female rat, ERα was present in synaptic vesicles in the axon of some GABAergic basket cells; 17β-E moved these ERα-containing vesicles toward synapses within 24 h (36). In addition, some proportion of this extra-nuclear ERα at synaptosomes and vesicles was phosphorylated though the function of phosphorylation in localization of the ERα remains unknown (37). In hypothalamic neurons and astrocytes obtained from both male and female rats, a full-length 66 kDa form of the ERα and a 52 kDa variant has been detected using surface biotinylation and immunocytochemistry (38, 39). These studies demonstrate that the nuclear ERα is present at the plasma membrane in many cell types, including the hypothalamus.

### Targeting of ERα to the plasma membrane

Though hydropathicity analysis shows that ERα may have a potential trans-membrane domain (38), the idea that ERα requires a membrane protein to tether it to the plasma membrane has been cemented primarily by the discovery of caveolin proteins that can anchor the ERα in lipid rafts and caveolae. Caveolin proteins are highly conserved structural proteins that are necessary and sufficient for the existence of caveolae, a subset of lipid rafts (40) that comprise a restricted compartment for signaling molecules associated with the plasma membrane. Initially, ERα was reported to interact with caveolin 1 (Cav-1) in MCF-7 and vascular smooth muscle cells (VSMC) (41). The caveolins in turn act as adaptor proteins to couple ERα selectively to either other proteins such as the metabotrophic glutamate receptors (mGluR) or to processes such as palmitoylation. In the hippocampus of the female rat, Cav-1 interacted with mGluR1a so that 17β-E activated downstream Gα_q_ and ERK signaling. This, in turn, increased pCREB activation within 5 min of 17β-E application (42). Hence, inhibitors to phospholipase C (PLC) and ERK and mGluR1a as well as a dominant negative Cav-1 mutant decreased pCREB activation (42). On the other hand, Cav-3 tethered ERα in hippocampal neurons to mGluR2/3; this pathway activated Gα_i_ signaling, leading to the inhibition of PKA and the subsequent downregulation of pCREB (42). Hence, the type of caveolin dictated the final divergent pCREB response though the physiological conditions wherein ERα may bind to Cav-1 versus Cav-3 are unknown. In hypothalamic astrocytes, the coupling of ERα to mGluR1a resulted in an increase of calcium within 2 min of 17β-E application; this was blocked both by the ERα and ERβ antagonist, ICI 182,780 and by the mGluR1a antagonist LY 367385 (43). In striatal neurons, Cav-1 facilitated the tethering of ERα to mGluR5 and subsequent Gα_q_ signaling (44). Hence, caveolins may not only tether ERs into caveolae in the plasma membrane in different cell types but also subserve tissue-specific signaling to specific Gα subunits by co-opting different mGluR partners. The coupling of ERα to mGluRs may allow downstream signaling to be potentiated by rendering it sensitive to two ligands, i.e., 17β-E for the ER and glutamate for the mGluR. This is supported by the data that in hypothalamic astrocytes, glutamate, and 17β-E combined elicited greater Ca^+2^ increases than either ligand alone (43).

In addition to the role that mGluRs play in linking ERα to Gα proteins, ERα can also directly bind Gα subunits. 17β-E inhibited cAMP production within 5 min of addition to GT1–7 immortalized GnRH neurons via ERα that is physically tethered to Gα_i3_ (45) protein that can be detected in co-immunoprecipitation experiments. Interestingly, addition of 17β-E decreased the amount of membrane-associated Gα_i3_ and this decrease was blocked by the ER antagonist ICI 182,780 suggesting control of the signaling cascade by the ligand, similar to classical G-protein coupled receptors (GPCRs) (45). Though ERα has been demonstrated to tether to Gα_i_ in the caveolae of endothelial cells to increase the activity of nitric oxide synthase (46), this mode of regulation of nitric oxide synthase by 17β-E has not been shown in the CNS.

**Figure 1:**
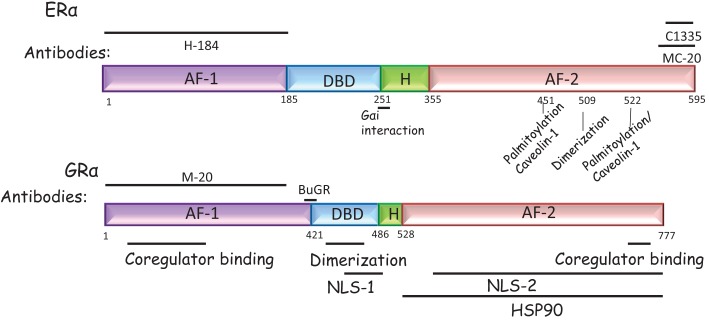
**Domain structure of the ERα and the GRα**. ERα and GRα are classical intracellular nuclear receptors but may also be present on the plasma membrane. Both receptors have a N-terminal AF-1 domain, a central DNA binding domain, a hinge domain (H), and a C-terminal AF-2 domain, required to bind ligand. ERα: the antibodies most commonly used to detect ERα are the H184 (Santa Cruz Biotechnology, TX, USA) antibody raised to the N-terminal domain and the MC-20 (Santa Cruz Biotechnology, TX, USA) and C1335 (Upstate Biotechnology, NY, USA) raised to the C-terminal domain. In addition, the sites that may tether the ERα to the plasma membrane are marked. These are residues required for caveolin or Gα_i_ binding or residues that are palmitoylated or required for dimerization at the membrane. GRα: the antibodies most commonly used to detect GRα are the BuGR antibody (Abcam Inc., MA, USA) and the M-20 (Santa Cruz Biotechnology, TX, USA) antibody, directed to the N-terminal domain. The NLS refers to the nuclear localization signal since nuclear localization of this receptor can occur rapidly and is a non-genomic effect.

Apart from serving as adaptors, the binding of caveolins to the ER also increases palmitoylation of the ER, a process by which palmitate, a C16 fatty acid is added to an internal cysteine via a thioester bond. In the human ERα, a canonical palmitoylation site exists at the cysteine amino acid residue at position 447 (C447); the corresponding site in the mouse ERα is the C451 (31). Mutant ERα where the C447 site was mutated to alanine (C447A) did not bind Cav-1, was not localized at the membrane in HeLa cells and showed decreased pERK activation in response to a 10-min application of 17β-E (47). Additionally, when a non-classical palmitoylation site, S522, in the ERα was mutated, localization at the plasma membrane and Cav-1 binding to ERα was decreased (48) in Chinese hamster ovary (CHO) cells. This mutant also decreased the ability of 17β-E to increase pCREB in hippocampal neurons from the female rat (42). Other binding partners for the ERα also lead to increases in palmitoylation and subsequent membrane localization. For example, the amino acid residue cysteine at position 451 of the mouse ERα also binds the heat-shock protein 27 (hsp27), which increased the rate of palmitoylation at this site. Therefore, siRNA to hsp27 decreased palmitoylation with subsequent decrease of ERα at the plasma membrane and decreased phospho-Akt (pAkt) normally induced by a 10 min application of 17β-E (49). The C-terminal of mouse ERα (Leu512–514) is important in ERα dimerization at the plasma membrane; mutation of these residues decreased pAkt and cAMP generation within 5 min of 17β-E addition and decreased the activation of both Gα_s_ and Gα_q_ subunits in the CHO cell line (50) (Figure 1). In the CNS, it is unknown if the ERα at the membrane exists as dimers and if these dimers use caveolin proteins as adaptors. It is also unclear if caveolin binding to the ERα precedes greater palmitoylation since ERα mutations that destroy the interaction with caveolin also destroy palmitoylation. Recently, E2-BSA has been shown to transcriptionally upregulate Cav-1 via a PI3K and ERK pathway within 12 h in endothelial cells (51). Hence, 17β-E may increase non-genomic signaling both by increasing the palmitoylation of ERα within rapid time frames and within longer time frames via the transcriptional upregulation of Cav-1.

### Regulation of ERα at the membrane by 17β-E

Similar to the tissue specificity shown by ERα-mediated rapid, non-genomic signaling, ERα at the membrane is also regulated by 17β-E in both temporal and tissue-specific manner. In hypothalamic astrocytes (38) and neurons (39) and in a hypothalamic cell line, mHypoE-38 (52), 17β-E increased the amount of both the 52 and 66 kDa mER in the cell membrane within 30 min. This increase of mERα at the plasma membrane can be blocked by ICI 182,780 and the mGluR1a antagonist LY 367385 but does not occur with E2-BSA, suggesting that this is an internal, non-membrane-initiated rapid effect that is dependent on the mGluR1a receptor. In contrast, in MCF-7 cells, 17β-E decreased Cav-1 within 8 h, thus decreasing the amount of ERα at the membrane. Similarly, 17β-E treatment of hippocampal slices from female rats removed ERα from the synaptosomal plasma membrane by depalmitoylation within 20 min (37). In CHO cells, palmitate incorporation into ERα and association with Cav-1 decreased within 60 min of 17β-E administration, suggesting that ERα at the plasma membrane was lowered, consistent with this idea, ERK activation also decreased (47). Decreasing ERα at the membrane in these cells may allow for a decrease in non-genomic signaling and may mark a shift toward genomic transcription. In contrast, in the VSMC, 17β-E increased both Cav-1 and Cav-2 and increased ERα localization at the plasma membrane (41). Such increased ERα localization in response to the endogenous ligand may be a mechanism to sustain non-genomic signaling in some cell types.

Similar to other trans-membrane GPCRs, which may undergo desensitization via internalization in a G-protein receptor kinase 2 (GRK2)-β-arrestin-dependent manner (18), 17β-E application to cortical neuronal cultures rapidly induced GRK2 phosphorylation within 5 min and β-arrestin1 and c-Src recruitment to the ERα within 60 min. Predictably, since β-arrestin1 tagging of trans-membrane GPCRs provides a scaffold for c-Src attachment and subsequent ERK activation (53), siRNA to β-arrestin1 abrogated the 17β-E induction of pERK in these neurons (54). Though the exact partners of the ERα in this internalized signalosome are unknown, ERα internalization is initiated from the membrane since E2-BSA also promotes internalization (54). Internalization of the ERα has also been tested more directly by using a surface biotinylation technique followed by stripping the surface biotin with glutathione after 17β-E addition to cells. Hence, the continued presence of biotin in this preparation would argue that the receptor was internalized and protected against glutathione action. In hypothalamic neurons, addition of 17β-E caused internalization of both the 52 and 66 kDa forms within 30 min, with internalization of the 52 kDa form persisting for 2 h (54), demonstrating that recycling and internalization of the classical ERα as well as the 52 kDa variant occurs in a rapid time frame. An unresolved issue is the rapid decrease in total ERα protein and mRNA in the hypothalamus on 17β-E application that has been shown by several investigators (55, 56). However, it is difficult to reconcile this with the increase in plasma mERα that is seen in the hypothalamic primary neuronal and astrocytic cultures (38, 39) and in the hypothalamic cell line mHypoE-38 (52) unless ERα from other non-membrane locations decrease to a large extent; this could be a parameter to be investigated in future studies. This could also be due to the recent studies that show an increase ERα at the plasma membrane being carried out in primary cultures versus hypothalamic tissue that retain neuronal connectivity in the older studies.

### Dependence of rapid signaling on ERα and ERβ

In addition to direct demonstration of the ERα either at the membrane or tethering to an intrinsic membrane protein, the ER dependence of kinase activation has also been investigated. Several studies, described in this review, have used the ERα and ERβ antagonist ICI 182,780 to antagonize a rapid signaling pathway or a genetic approach that deletes either ERα or ERβ specifically in the whole animal. The availability of ERα and ERβ knockout animals, i.e., ERαKO and ERβKO, has been useful to understand the ER dependence of 17β-E-mediated non-genomic signaling *in vivo*, particularly in light of an absence of a specific antibody to the ERβ (57). In wild-type female mice, subcutaneous administration of 17β-E caused the activation of CREB and ERK within an hour, as measured by immunohistochemistry for the phosphorylated forms of these molecules. When either ERα or ERβ or both were deleted, a complex brain nuclei-specific and isoform-specific ER dependence was revealed, which may in part be due to the tissue distribution of the ER isoforms in the brain (Table 1 below) (58). For example, in the ventrolateral VMH (vl-VMH), the loss of ERα results in the reduction of pCREB on 17β-E administration and this could be because the predominant ER isoform is the ERα and it is possible that the loss of ERα cannot be compensated by smaller amounts of ERβ (58). The use of ERαKO, ERβKO, and ERαβKO mice in this study showed that ERα is hence both necessary and sufficient for the induction of pCREB. In the mPOA, 17β-E could induce pCREB via either ERα or ERβ but required both ERα and ERβ to act in conjunction in order to induce pERK (58), demonstrating that ER isoforms can regulate rapid signaling differentially in the same tissue. The regulation in male mice is unknown and is under investigation in our laboratory.

**Table 1:** **Comparison of CREB and ERK between wild-type (WT), ERα knockout (ERαKO), ERβ knockout (ERβKO), and double ERα ERβ knockout (ERαβKO) female mouse brain**.

Genotype	vlVMH		mPOA		Medial septum	
	pCREB	pERK	pCREB	pERK	pCREB	pERK
WT	++	NR	++	++	++	NR
ERαKO	−	NR	++	−	++	NR
ERβKO	++	NR	++	−	−	NR
ERαβKO	−	NR	−	−	−	NR

*pCREB and pERK in 17β-E injected mice was quantitated by immunohistochemistry and compared to the levels of pCREB and pERK present in mice injected with the vehicle, in the ventrolateral VMH (vl-VMH), the medial preoptic area (mPOA) and the medial septum in female ovariectomized mice injected with 10 μg 17β-E and sacrificed an hour after injection. In WT mice, 17β-E increased pCREB in the vl-VMH and pERK and pCREB in the mPOA. ER isoform dependence varies according to the output (pCREB or pERK) measured and the nuclei. ++: upregulation compared to vehicle-treated mice. −: no upregulation compared to vehicle-treated mice. NR = data not recorded. Data are from Ref. (58)*.

### ERα variants

In both hypothalamic neurons and astrocytes (38), a variant 52 kDa ERα (ERα-52), detected with a C-terminal antibody that also detects the full-length 66 kDa, was expressed in much higher amounts than the ERα-66 kDa form. Based on RT-PCR analysis using flanking primers to this exon in the mouse hypothalamic cell line mHypoE-38, this is believed to be an ERα that lacks exon 4 (ERαΔ4) (52). In astrocytes, only the 66 kDa form is thought to be interact with mGluR1 (43) and the interaction, signaling properties, and function, if unique, of the 52 kDa variant remain unknown. A 36 kDa variant lacking the AF-1 and AF-2 domains with a unique C-terminus was detected in human breast cancer cells, coded for by a unique promoter located in intron 1 of the *Esr1*gene (59) and can be detected by a specific antibody. ERα-36 was localized to the plasma membrane, mediated rapid ERK signaling, and is induced by the selective activation of the GPR30 (discussed in Section “GPR30”) by the agonist, G-1 (60). Importantly, this GPR30-selective activator, G-1, has also been demonstrated to bind and activate ERα-36 (60), suggesting that some of the responses attributed to the GPR30 might be via ERα-36. A 46 kDa variant observed in endothelial cells is also localized to the plasma membrane (61) and is composed of a splice variant missing the first 173 amino acids of ERα-66 (62). ERα-46 cannot be detected with an N-terminus antibody raised to ERα-66 such as H-184 (Figure 1), but should be identifiable with a C-terminus ERα antibody. A novel ER is the ER-X, an ER of 63 kDa that is expressed in the neocortex in the caveolae membrane fraction, can be identified with ERα and ERβ antibodies and is present in the ERα knockout animal (63). This ER, whose structure remains unidentified, mediated ERK signaling by both 17α-estradiol and 17β-estradiol and would not meet the criteria for a mER, based on our previous definition. The function and expression pattern of the ER-X is unknown though it was upregulated after ischemic injury (63) in the adult. If it is not present in the adult hypothalamus, it is unlikely to be a receptor that mediates normal behavior that is dependent on the hypothalamus (63). So far, neither ERα-36 nor ERα-46 has been demonstrated in the CNS, though it is possible that some antibodies directed toward the DNA-binding domain or, in the case of the 46 kDa variant, the C-terminal domain of the full-length 66 kDa ERα such as MC-20 or C1335 (Figure 1) may detect these forms in studies that use solely immunocytochemistry.

### Gα-coupled mERs

The idea that the mER is a Gα_q_-coupled receptor has been explored in the POMC neuron in the ARH, primarily by the laboratories of Kelly and Ronnekliev (7, 64, 65). In POMC neurons, 17β-E and E2-BSA uncoupled the inhibitory μ-opioid receptor from the G-protein coupled inward rectifying K^+^ (GIRK) channel, in a PLC-, PKC-, and PKA-dependent manner (66, 67), thus decreasing hyperpolarization of these neurons and increasing neuronal excitability. Pharmacological characterization revealed that a Gα_q_ receptor upregulated PLC and calcium release, which in turn activated PKC and PKA. Subsequent PKA phosphorylation of the GIRK channel uncoupled the μ-opioid receptor (7, 68) from GIRK. Though this response was specific for 17β-E versus 17α-E and was blocked by the ERα and ERβ antagonist ICI 182,780, it also occurred in ERαKO, ERβKO, and GPR30 KO mice (69), suggesting that a novel mER is coupled to Gα_q_. This signaling pathway can be activated by a selective ligand, STX, which does not bind ERα, ERβ, or GPR30. Both STX and 17β-E also reduced post-ovariectomy weight gain, suggesting that this novel Gα_q_-coupled mER is important in energy homeostasis. Leptin receptors on POMC neurons caused depolarization rapidly via the JAK/STAT and PI3K pathways that open calcium-dependent TRPC channels (70); 17β-E signaling may synergize with leptin action by increasing calcium rapidly in these neurons via this Gα_q_-mER. Several reviews (7, 68, 69, 71–74) detail the studies on this STX-activated receptor.

### GPR30

The GPR30, also known as GPER1, is a former orphan GPCR that was shown to bind 17β-E and increase cAMP in breast cancer SKBR3 cells via an increase in adenylyl cyclase activity (75). In the rat hypothalamus, GPR30 expression is particularly high in the PVN and SON (76) with low expression in the VMH. Cell fractionation and immunocytochemistry revealed GPR30 to be localized at the plasma membrane in both the SKBR3 (GPR30^+^, ERα^−^, ERβ^−^) and MDA-MB-231 (GPR30^−^, ERα^−^, ERβ^+^; GPR30 overexpression) breast cancer cell lines (77). Though GPR30 was not detected at the plasma membrane using surface biotinylation in hypothalamic astrocytes (38), GPR30 was localized to the post-synaptic density in the hippocampus and associates with PSD-95 through its C-terminal tail (78). This association with PSD-95 can localize GPR30 to the plasma membrane, independent of 17β-E. Immuno-electron microscopy analysis of rat hippocampi also revealed membrane localization with no intracellular staining (79). However, it can be detected intracellularly in normal mammary gland epithelial cells (80) and colocalized with a marker of the Golgi apparatus in primary cultures of the hippocampus (81). Also, overexpressed GPR30 in COS7 cells exhibits localization at the endoplasmic reticulum and the Golgi (82), suggesting that cell type can determine localization. In addition, HeLa cells transfected with FLAG-GPR30 show mostly staining at the membrane while cells transfected with GFP-GPR30 show staining mainly in the endoplasmic reticulum [(79) #6908], suggesting that inclusion of molecular tags may interfere with proper intracellular trafficking, possibly confounding the interpretation of experiments relying on ectopic expression of GPR30.

Studies describing the regulation of internalization of GPR30 have also yielded conflicting results. When FLAG-GPR30 was ectopically expressed in HeLa cells, receptor endocytosis was ligand dependent and internalized GPR30 occupied a diffuse cytoplasmic localization consistent with classical receptor recycling or proteasomal degradation pathways (79). In human embryonic kidney (HEK)-293 cells, GPR30 internalization was not only ligand-dependent but also occurs constitutively (83). While most GPCRs are either recycled to the plasma membrane via endosomes or degraded in lysosomes to limit excessive signaling, GPR30 rapidly accumulates in the perinuclear compartment via clathrin-coated vesicles (83). It is thought that this Rab 11-dependent accumulation is in the trans-Golgi network and degradation occurs via an ubiquitin-proteasome-mediated pathway, unlike the endosomal degradation or recycling to the surface that is typical for other GPCRs (83). Since this pathway for internalization and degradation is rapid and constitutive, Cheng et al. propose this as the reason for the difficulty in detecting GPR30 at the plasma membrane in some cell lines (83).

GPR30 can signal via both the Gα_s_ and the G_βγ_ subunits that are associated with the receptor upon activation. The Gβγ subunit transactivated the EGFR receptor leading to downstream activation ERK protein, while simultaneous activation of the Gα_s_ subunit by 17β-E inactivated ERK signaling through activation of adenylyl cyclase and PKA, thus limiting cAMP signaling to a short time frame (23). Furthermore, although GPR30 protein was readily detectable in both the microsomal and plasma membrane subcellular fractions of breast cancer cell lines, only GPR30 in the plasma membrane fraction bound ligand and activated G-protein signaling, suggesting that only membrane-associated GPR30 protein is functional (77). However, GPR30 activation in COS7 cells initiated intracellular calcium mobilization and nuclear accumulation of PIP3 via EGFR transactivation (82) via a non-membrane-initiated signaling mechanism since calcium flux was not replicated by membrane restricted estradiol derivatives (84). These discrepant results have yet to be reconciled. A recent study reported that GPR30 could decrease cAMP that was elevated by heterologous ligands, such as forskolin, via the C-terminal PDZ domain (85). This domain could bind membrane-activated guanylate cyclases (MAGUKs), which act as adaptors for AKAP5 (PKA anchoring protein) that in turn decreased adenylate cyclase activity. This would decrease the cAMP that is elevated by 17β-E binding of the GPR30 and may serve to limit the time frame of GPR30 signaling (85). Hence, both ERK and cAMP signaling downstream of the GPR30 may be restricted to very short time frames.

### The iGR as the mGR

Similar to the ERα being considered a possible mER, the intracellular GR (iGR) that exists as a complex with heat-shock protein 90 (HSP90), p23, and a tetratricopeptide protein (86) in the cytoplasm has been proposed as a candidate mGR (87). Stable reduction of mGR levels in CD14^+^ monocytes using stably transfected siRNA to iGRα suggested that both mGR and iGR were derived from the same transcript (88). GR exists in two isoforms, GRα and GRβ. GRβ, generated by alternative splicing, lacks the last 50 amino acids of the GRα carboxy terminus, and instead possesses a unique 15 amino acid sequence at its C-terminus (89). GRβ neither binds glucocorticoids nor has intrinsic transcriptional activity, but has been implicated as a dominant negative inhibitor of GRα activity through formation of a non-functional heterodimer (90). Higher concentrations of GRβ relative to GRα, therefore, result in decreased glucocorticoid sensitivity. However, levels of GRβ protein in the brain are extremely low (89) and this is not thought to be a regulator of GRα in the brain. The presence of mGRα was detected at very low levels in human lymphocytes and leukocytes using membrane-impermeable fluorescent liposomes and GR-specific antibodies (91) such as M-20 (Figure 1). Similar to the tethering of ERα to the plasma membrane, mGRα has been associated with Cav-1 in MCF-7 cells (92). However, while plasma membrane association of other steroid hormone receptors, including ER, is dependent on palmitoylation, mutation of the homologous sequence in GR did not affect membrane localization (93), suggesting that other mechanisms must tether mGR to the plasma membrane. In the brain, most of the rapid effects of glucocorticoids have been confined to the hippocampus and to the hypothalamus. Membrane glucocorticoid receptors were first observed in the synaptic plasma membrane fractions (SPM) of rat brain via [3H]-corticosterone binding assays (20). Hypothalamic SPM had a higher binding capacity than hippocampal or cortical SPM, which is in contrast to corticosterone binding of the iGR, which is lower in the hypothalamus and much higher in cerebral cortex and hippocampus (94). Synaptosomal fractions from rat hippocampus contained plasma membrane-associated GR (95) and GR immunoreactivity was observed at the plasma membranes and vesicle membranes of the hypothalamus (96) while in the CA1, GR was shown in the spine (97). Functionally, JNK and p38 MAPK were activated within 10 min of glucocorticoid administration in primary hippocampal neurons (98). The hypothalamo–pituitary–adrenal (HPA) axis is also subject to rapid negative feedback at the level of the hypothalamus (99). Bath application of glucocorticoids to rat hypothalamic slices caused a rapid suppression of glutamate-mediated excitatory synaptic currents onto CRH neurons that was decreased by a CB1 receptor antagonist (13). The release of endocannabinoids that bind to the CB1 receptor is dependent on corticosterone rapidly acting on the CRH neuron and is dependent on Gα_s_-driven PKA activation in the CRH neuron (100). However, the release of nitric oxide (NO) that increased GABAergic inhibition (101) onto the CRH neuron was dependent on Gβγ signaling (100). Hence, in the PVN, rapid negative feedback by glucocorticoids on post-synaptic CRH neurons is exerted by a combination of suppression of presynaptic glutamatergic neurons and excitation of presynaptic GABAergic neurons. The identity of the mGR that can signal via both Gα_s_ and Gβγ subunits in the CRH neuron remains unknown.

## Unresolved Questions: mER and mGR

As is evident in the preceding sections, several questions about the identity of the mER and mGR remain. Though the strongest evidence exists for a post-translationally modified classical ER, there are other viable candidates for the ER (102) based both on evidence of proteins that interact with antibodies raised to the classical ER or to the continued existence of non-genomic effects in ERαKO and ERβKO mice (63). Different ER proteins might be mERs in different tissues (14) or different proteins might be mERs in the same tissue at different times to generate divergent rapid signaling outcomes within the same cell or different tissues that is congruent with incoming stimuli. It is also possible that the time frame of non-genomic signaling might be different when there are different mERs present – a GPCR such as GPR30 may activate the ERK and PKA pathways very transiently whereas ERα or ERβ at the membrane may be capable of more sustained activation. Again, this would change the response of the cell to stimuli, depending on the mER present. Second, if the mER is the ERα, the mechanism by which only a small proportion of the total ERα is targeted to the membrane is not known and strengthens the idea that different pools of ERα may exist within cells (30) with different functions. The rationale for the variable amount of ERα at the membrane in some cells versus others in cell lines is also unclear but could allow for 17β-E to employ non-genomic versus genomic signaling to different extents in different cells, which maybe at different physiological states. Third, apart from the difficulty in studying ERβ localization or interaction with other proteins due to the unavailability of a reliable antibody (57), the localization of the ERα variants is also particularly understudied, but the predominance of the 52 kDa form as opposed to the full-length 66 kDa form in the CNS (39, 52) argues for an important role of these variants in estrogen signaling in the brain, as opposed to other classical estrogen-responsive tissues such as the uterus. The rapid actions of these different ERα variants may also oppose each other in some cases (39). It is, therefore, likely that the ratio of different ERs, including mERs expressed in each cell that determines the cellular response to 17β-E exposure. The significance of these spatially separated and functionally opposing ER populations may be to modulate the hypothalamic and hippocampal neuron response to estrogen and prevent runaway signaling. Fourth, though the GPR30 has a role to play in estrogen-mediated physiology, the acceptance of GPR30 as a mER has not been universal (103) with some investigators preferring to term it as a “collaborator” to the ERα, which is deemed to be the primary mER that signals from the membrane (30). If GPR30 or Gα_q_-coupled novel proteins are the mERs in some tissues, it is also possible that they crosstalk with classical ERα or ERβ, accounting in some studies for the ICI-mediated antagonism of the effect. Though this cooperation between GPR30 and ERα has been shown to have a proliferative effect on ovarian cancer cells (104), a functional effect has not been demonstrated in the hypothalamus. In the ventral hippocampus in male mice, Hart et al. showed an injection of G-1 could increase the phosphorylation of an ERK-sensitive serine site on the ERα at position 118 within 30 min, suggesting that these two receptors could interact with each in the CNS (105). Future studies on the colocalization and the functional interaction between ERα and GPR30 in the hypothalamus will prove useful in this regard. There are far fewer studies on the mGR, when compared to the mER, and most studies investigate the possibility of the mGR being equivalent to the iGR. Surprisingly, the idea that different G-protein subunits, particularly that the Gβγ subunit can support signaling from the unidentified mGR in the CRH neuron and from the Gα_q_-coupled, STX-activated mER in the POMC neuron is also seen with a traditional GPCR such as GPR30. Whether signaling from both G-protein subunits is concomitant or if one signaling pathway predominates, under certain physiological conditions, is not known.

Lastly, the model generated by the existing literature is that the mER or mGR is at the inner leaflet of the membrane, associated with intrinsic membrane proteins or scaffolds such as caveolin. However, surface biotinylation experiments imply that some part of the mER or mGR is exposed to the extracellular mileu (18). Though an abundance of hydrophobic amino acids in the ligand-binding domain of the mER predicts that this domain might insert into the plasma membrane (106), no study has demonstrated this in the CNS. While recycling of this receptor from the membrane is to be expected, the pathways (Rab-mediated or β-arrestin mediated) are possibly cell-specific and are worthy of more attention since non-genomic signaling is possibly terminated during internalization.

## Morphological and Behavioral Outputs in the CNS Dependent on Non-Genomic Signaling by Estrogens and Glucocorticoids

Though kinase regulation and calcium flux has been shown to occur rapidly when cells are exposed to the hormone in a number of tissues and the idea of non-genomic signaling more accepted than ever before, the relevance of non-genomic signaling for behavior has remained murky. Here, we shall confine ourselves to primarily hypothalamically driven behaviors or possible neural correlates that are either rapidly induced by 17β-E or E2-BSA or that may have a non-genomic component. Finally, we will discuss the intersection of glucocorticoid rapid signaling in a 17β-E-mediated behavior.

### Spinogenesis

Estrogen is critical in the display of female sex behavior in rodents by acting on the hypothalamus; retrograde tract tracing using the pseudorabies virus (PRV) injected into the lumbar muscles labeled the lordosis circuit, in particular, the plexus of oxytocin fibers in the vl-VMH (107). In the hypothalamus, estradiol benzoate (EB) injections to ovariectomized rats increased spine density by 48% in the vl-VMH as compared to oil injections (108). Surprisingly, only 3% of PRV-labeled neurons were ERα^+^ and these were not the neurons that showed an increase in spine density (109) upon EB administration. These data argue that the increase in spine density in the VMH may be only indirectly dependent on ERα signaling or could be due to another ER while the longer time frames used in the study do not allow us to conclude if there is a non-genomic component. A candidate could be the GPR30 receptor, which is expressed in the vl-VMH; the role of this receptor in spinogenesis in the hypothalamus is not known. Rapid effects on spinogenesis and spine morphology following GR activation have been observed in the hippocampus, but not in the hypothalamus. CA1 neurons from male rat hippocampal slices treated with dexamethasone for 1 h demonstrated a translation-independent increase in spine density, which was lost with co-application of dexamethasone with either the GR antagonist RU-486 or the NMDA receptor blocker MK-801 (95). The proportion of mushroom-shaped and thin-type spines was also increased following GR activation (95).

Does non-genomic signaling play a role in the increase in spine density by estrogens or by glucorticoids? The mGluR1a antagonist, LY367385 in the ARH decreased phosphorylated cofilin levels that are induced within an hour of EB injection and that are required for actin reorganization and spinogenesis, suggesting that at least some of the initial aspects of spinogenesis are mediated via non-genomic signaling from the ERα-mGluR1a complex at the membrane (110). Though EB can increase filopodial spines in the ARH within 4 h of treatment, mushroom shaped, more stable spines require time frames in excess of 20 h and parallels the time frames required for the full display of lordosis behavior, suggesting that rapid non-genomic signaling is insufficient for formation of stable spines (110). Stabilization of the PSD-95 protein, a scaffolding protein enriched in mushroom-shaped dendritic spines, was dependent on pAkt and also required 48 h of 17β-E treatment in differentiated NG-108 cells (111). Another model for the increase in 17β-E-mediated increase in spine density in the hypothalamus is that 17β-E stimulates PI3K activation pre-synaptically, inducing glutamate release, followed by NMDA receptor activation and ERK signaling post-synaptically, with a subsequent increase in spinophilin protein that was correlated with an increase in stable dendritic spines (112) on the post-synaptic neuron. Both ERK and PI3K signaling are implicated in the increase in spine density, though the formation of mature mushroom spines also appears to require transcription (113). Though there are no reports of glucocorticoid-mediated regulation of spine density in the hypothalamus, suppression of PKA, PKC, MAPK, or PI3K signaling completely blocked GR-mediated spinogenesis in CA1 neurons, suggesting that GR signals through convergent kinase pathways to increase actin polymerization, which would allow for spine changes (114). In CA1 neurons, treatment with the synthetic glucocorticoid dexamethasone increased p-cofilin levels within 30 min, similar to the rapid induction seen in the ARH by EB (97).

Consistent with the idea that 17β-E-mediated spinogenesis in the VMH has a functional consequence, cytochalasin D, an actin polymerization inhibitor, blocked the formation of spines in the ARH and reduced lordosis (110) though the relevance of such spinogenesis to sex behavior in rodents (see Sex Behavior or Lordosis in Females) is unknown. The consequences of spine disruption for other behaviors such as aggressive behavior or male sex behavior (see Male Sex Behavior) are also unknown. Though most of the studies have shown effects on spine density or morphology in longer time frames that do not allow one to parse non-genomic actions from transcription, spine density on the apical dendrite in the stratum radiatum and in the stratum lacunosum moleculare of the CA1 neuron was increased within 40 min by the ERα selective agonist, propyl pyrazole triol (PPT); however, these were at high doses that were not correlated with doses of PPT that led to an improvement in social memory within 40 min of administration (115). In mature rat cortical neurons, 17β-E rapidly increased ERK and p21-activated kinase to increase dendritic spine density (116) within 30 min. Though this suggests that non-genomic signaling may be sufficient for an increase in spine density at least in the hippocampus and cortex, this has to be confirmed with experiments that utilize a transcription inhibitor. A similar rapid effect of estrogen on spine density in the hypothalamus has not been demonstrated.

### Sex behavior or lordosis in females

Lordosis in rodents is a 17β-E-driven behavior where integration of sensory information within the limbic-hypothalamic circuit culminates in VMH projections onto neurons of the periaqueductal gray (PAG) and spinal cord motor neurons, resulting in the classical lordosis posture (16). A series of elegant experiments from the laboratory of Micevych delineated a microcircuit consisting of neuronal afferents from the ARH to the mPOA (113). In this circuit, the initial membrane-initiated signaling by 17β-E in the ARH neurons increased PKCθ (117) and released neuropeptide Y that activates β-endorphin expressing ARH neurons that project into the mPOA (118). In the mPOA, β-endorphin released from the ARH caused the internalization of μ-opioid receptors (MOR) within 30 min of E2-BSA (119) administration into the ARH, suggesting that this is a membrane-initiated non-transcriptional event. This transient inhibition of lordosis initiated by MOR internalization is removed 30 h later by progesterone (120). A number of different receptors, including ERα and mGluR1a, play a role in this membrane-initiated signaling to influence lordosis behavior. Not only does ERα and mGluR1a colocalize in about 23% of ERα-expressing neurons but deletion of ERα also blocked MOR internalization in the mPOA (121) and abolishes lordosis (122). Blocking of MOR internalization with application of an mGluR1 antagonist or Cav-1 siRNA into the ARH also, in turn, reduced lordosis (119, 123). Though this suggests that ERα and mGluR1 signal together as in the hippocampus (124), the selective mER agonist, STX in the ARH also induced MOR internalization and lordosis (125), an effect blocked by the mGluR1a antagonist. This suggests that in the ARH, both ERα and the Gα_q_-mER may couple to mGluR1a and facilitate lordosis, though the mechanisms by which this occurs are unclear. Apart from these receptors, the localization of the GPR30 in the hypothalamus (126) and the ability of G-1, an agonist at the GPR30 to facilitate lordosis in female ovariectoimized mice (127) argues that GPR30 signaling is also important, at least in mice. It is unknown if the ERα, the Gα_q_-mER, and the GPR30 interact or signal to each other or represent independent parallel signaling pathways that drive lordosis in estrogen primed ovariectomized female rodents, though any model must take into account the necessity of ERα in the VMH for lordosis.

Since Kow et al. demonstrated that E2-BSA alone in the VMH does not induce lordosis (128), we can presume that non-genomic signaling is insufficient but may potentiate or prime lordosis that is in itself dependent on nuclear transcription (129) via a coupled signaling pathway (130). Consistent with this idea, a mouse that possesses an ERα that cannot bind an ERE (ERα^−/AA^) (131) did not display lordosis behavior (132), demonstrating the importance of 17β-E-bound ERα transcriptional action at an ERE for lordosis. Also, combined E2-BSA and 17β-E administrations into the VMH increase progesterone receptor (PR) protein levels higher than either hormone alone (133), demonstrating potentiation of transcription by a membrane-limited estrogen conjugate. The reduction of ERα in the VMH using adenovirus-associated shRNA viral vectors that abolished lordosis behavior (29) shows the absolute requirement for the ERα, which could be due to its participation both as a mER and as a nuclear transcription factor in the VMH. An additional role could be the signaling initiated by ERα acting as a mER in the ARH but as a transcription factor that upregulates the PR in the VMH (134), both processes that are required for lordosis. In this scenario, membrane-initiated signaling in the ARH results in transient inhibition of lordosis so that 17β-E can transcriptionally activate genes such as the PR in the VMH that are important for the full display of lordosis that occurs 30 h later (113).

### Male sex behavior

In males, 17β-E given intraperitoneally at high concentrations to castrated adult rats increased some aspects of male sex behavior, i.e., genital sniffs and mounting within 15 min, a time frame commensurate with non-genomic actions (135). However, since protein synthesis inhibitors (136) and the presence of the ERE-binding mutant, the ERα^−/AA^ mouse abolished male sex behavior (131), it is reasonable to conclude that male sex behavior, similar to female sex behavior, has a non-genomic component that is not sufficient for the full display of the behavior. Though most of the studies investigate the role of testosterone in males (137, 138), testosterone can be converted to 17β-E by aromatase (139). In the castrated and testosterone supplemented quail that was treated with the aromatase inhibitor, vorozole, administration of 17β-E or E2-BSA intracerebroventricularly (icv) increased appetitive sexual behavior but not the final consummatory aspects of sexual behavior within 15 min (140). This appetitive sexual behavior was also blocked by vorozole, administered icv 15 min before behavioral testing (140). This implies that neuroestrogens that are rapidly generated in response to stimuli such as the sight of a female signal rapidly to initiate appetitive sexual behavior. Though ICI 182,780 antagonized the increase in appetitive sexual behavior seen rapidly with 17β-E administration (140), the exact mER that mediates the rapid regulation of sex behavior or aromatase in the quail or the mouse is unknown. In the male mouse, consistent with the interpretation that arises from the data in the quail, 17β-E administered to aromatase knockout male mice reversed the lack of sex behavior while vorozole decreased sex behavior in C57BL/6 mice within 15 min of administration (141). Stimuli-dependent rapid regulation of aromatase could represent a mechanism to locally elevate neuroestrogens, which then increase sex behavior in male quail or in the male rodent. However, contrary to this expectation, aromatase activity decreased rapidly when male quail were exposed to female stimuli but neuroestrogen concentrations itself increased in the mPOA (142), a nuclei important for male sex behavior. However, it is important to note that the acute action of 17β-E on appetitive sexual behavior in the male quail was always preceded by initial copulation with a receptive female to establish this learned response, wherein the sight of the same female would initiate appetitive sexual behavior (143). In this case, it is possible that prior sexual experience may initiate transcriptional signaling by 17β-E, which in turn primes rapid non-genomic signaling to achieve greater efficiency in subsequent sexual interactions and optimize reproduction; this remains to be tested. Similar to female sex behavior, the non-genomic component in male sex behavior is important for the initial approach and mounting aspects of male sex behavior.

### Aggression in males

Castration in male rodents removes both testosterone and estrogens and results in loss of aggression in response to territorial intrusion (144, 145). The importance of estrogen in male aggressive behavior is shown by the deletion of aromatase, which abolished aggression by male mice in a resident-intruder paradigm, though this is possibly also due to organizational defects since 17β-E reinstated aggression only when administered before post-natal day 7 (146). In CD-1 outbred male mice, ERα concentrations in several areas of the circuit involved in aggression such as the bed nucleus of the stria terminalis (BNST), the lateral septum (LS), and the anterior hypothalamus (AH) was higher in more aggressive mice (147). Reduction of ERα in the VMH by adenovirus mediated transfer of siRNA to ERα abolished aggressive behavior in male mice, demonstrating that ERα expression in the VMH is necessary for aggression in adult male mice (29), just as it was important for sex behavior in male mice. In California, beach mice that were castrated and supplemented with testosterone and an aromatase inhibitor so that they possessed testosterone but not estrogen, aggression increased rapidly within 15 min of injection with cyclodextrin-conjugated 17β-E (148). This suggests that testosterone alone was not sufficient to elicit aggressive behavior in a resident-intruder paradigm and that 17β-E is necessary to activate aggression. The time frame and the inability of cycloheximide (149) to decrease this aggression in the beach mouse argue that 17β-E acts non-genomically, though most studies on aggression, with the exception of those cited in this paragraph, in the rat and mouse have used time frames that do not allow us to parse a non-genomic effect from a genomic/coupled signaling effect.

What is the source of 17β-E? In rodents, aromatase is expressed in axon terminals (150–152) particularly in the hypothalamus (153) and brain 17β-E concentrations in the male rat are equal to or higher (~8 nM) than in the plasma (154). Hence, 17β-E generated at the synapse has been envisioned to function as a neuromodulator in short time frames (155), in line with the data in male birds where neuroestrogens generated at axon terminals are important in learning in songbirds (156) and in sex behavior in the quail (157, 158). In the castrated male rat, EB synergized with dihydrotestosterone (DHT) to increase aromatase activity (159). In addition, 17β-E can transcriptionally induce the aromatase gene via ERα-c-jun complex binding to AP-1 elements in the brain-specific promoter of aromatase (160). Therefore, similar to spinogenesis, the regulation of aromatase by 17β-E itself may represent another correlate where both rapid, non-genomic and slower, transcriptional mechanisms converge. The areas of the brain wherein this may occur in the rodent are not clear and are being investigated in our laboratory.

Though 17β-E can rapidly increase aggression by modulating neurotransmitter release [(145) and references therein], it may also do via regulating the HPA axis. In the white-crowned sparrow, neither brain 17β-E concentrations nor aromatase activity in nuclei involved in aggression were correlated with aggressive behavior (161) though corticosterone did increase rapidly. What is the link between glucocorticoids and aggression? Though aggression itself is stressful and leads to secretion of glucocorticoids, dominance in an aggressive encounter typically leads to lowering of the corticosterone level (162). However, pre-fight corticosterone levels have been shown to be associated with aggressiveness in fish and acute treatment with adrenocorticotrophic hormone (ACTH) increased fighting behavior in male mice (163) while acute treatment with corticosterone 2 min before an encounter increased aggressiveness in male rats (164) and risk assessment in the open field and elevated plus maze tests (165). Supporting the idea that corticosterone is important in regulation the onset of aggressive behavior, acute treatment with GR antagonists (166, 167) or prevention of glucocorticoid synthesis (164) prevented aggression when presented with social challenge. Since acute central application of corticosterone increased aggression within 7 min and protein synthesis inhibitors did not decrease it, non-genomic signaling by glucocorticoids in the CNS is most likely required to increase aggression (164). Consistent with this idea, acute corticosterone injection also decreased the magnitude of electrical stimulation to the hypothalamus required to elicit attack (168). In addition, corticosterone administration to rats in established colonies did not change aggression, demonstrating that glucocorticoids promote aggression only in ethologically relevant situations such as territorial intrusion (169). However, chronic elevated levels of glucocorticoids, such as seen in stressed or socially defeated animals decrease aggression and increased submissiveness in hamsters (170, 171), mice (172), and rats (173, 174). This is thought to be due to genomic signaling by glucocorticoids though the molecular mechanisms by which glucocorticoids facilitate or inhibit aggression are unclear (175).

**Figure 2:**
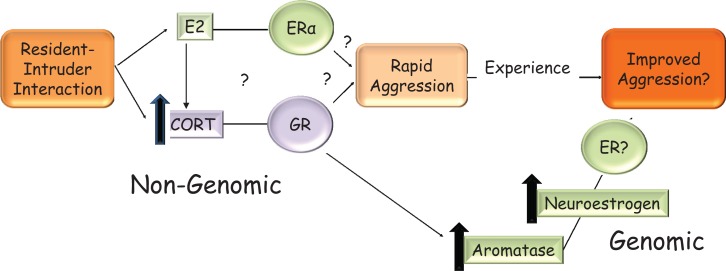
**Hypothetical model in the rodent for the rapid and sustained increase in aggression that is seen when an intruder enters a resident’s home-cage**. On visual and/or olfactory cues, 17β-E in the brain may increase corticosterone via an unknown mechanism. This increase in corticosterone may rapidly and non-genomically increase aggressive behavior. The mechanism by which corticosterone does so is not known but may involve the regulation of aromatase and could be GR mediated. It is not known if 17β-E’s regulation of aggressive behavior is solely via corticosterone increase or these are two parallel pathways, which require ERα, as shown in the figure. Elevated corticosterone levels can increase transcription from the brain-specific aromatase promoter, which in turn increases neuroestrogens. This may contribute to shorter latencies of attack in successive bouts. The interaction of estrogen and corticosterone ensures that the resident rodent is aggressive only in the presence of a relevant external cue, i.e., intruder and a relevant internal cue, i.e., sufficient 17β-E.

Gonadal status can modify the actions of glucocorticoids on the hypothalamus or can regulate corticosterone levels itself. For example, gonadal steroids can modify glucocorticoid-mediated negative feedback on CRH. 17β-E chronically administered to gonadectomized and adrenalectomized female rats treated with high doses of corticosterone increased CRH mRNA levels, thus counteracting negative feedback, while DHT treatment to male rats had an opposing effect and caused a further decrease in CRH levels (176). In female ovariectomized rats, icv, but not systemic, 17β-E injection increased corticosterone rapidly within 30 min, an effect mimicked with the ERα selective agonist, PPT, injected into the PVN (177). In gonadectomized male rats, CRH mRNA was higher when they were treated with EB but lower when treated with DHT, implying that estrogens can regulate the HPA axis in males also (178). A number of mechanisms may be involved in the rapid increase of aggression by glucocorticoids in response to social challenge, including the regulation of dopamine (179, 180) and serotonin (181, 182) neurotransmission. Here, we will focus on one such mechanism that is responsive to both estrogens and glucocorticoids in the hypothalamus, namely, the regulation of aromatase. Acute stressors, which increase glucocorticoids, increased neuroestrogen concentrations in the PVN and aromatase mRNA in the PVN within an hour in female rats, suggesting that corticosteroid release may also increase neuroestrogen in the hypothalamus (183). Similarly, in male quail, rapid increases in aromatase activity also occurred on restraint stress in the mPOA (184), demonstrating that corticosterone can affect aromatase using a non-genomic signaling mechanism that is yet unknown, in both birds and rodents. Furthermore, aromatase protein levels are increased in response to glucocorticoid treatment in a hypothalamic cell line via a transcriptional mechanism (185), leading to a possible increase in the amount of aromatase that may increase the efficiency of winning future bouts. These data demonstrate that 17β-E in the brain can increase plasma corticosterone, which in turn can increase aromatase activity and/or transcription in the hypothalamus leading to a positive feedback circuit that may be active under physiologically relevant stressful contexts such territorial intrusion. The relevance of this pathway (Figure 2) to aggression is under active investigation in our laboratory, with the idea that glucocorticoid control of aggressive behavior is dependent on neuroestrogen concentrations in the brain.

## Functions of Non-Genomic Signaling

**Figure 3:**
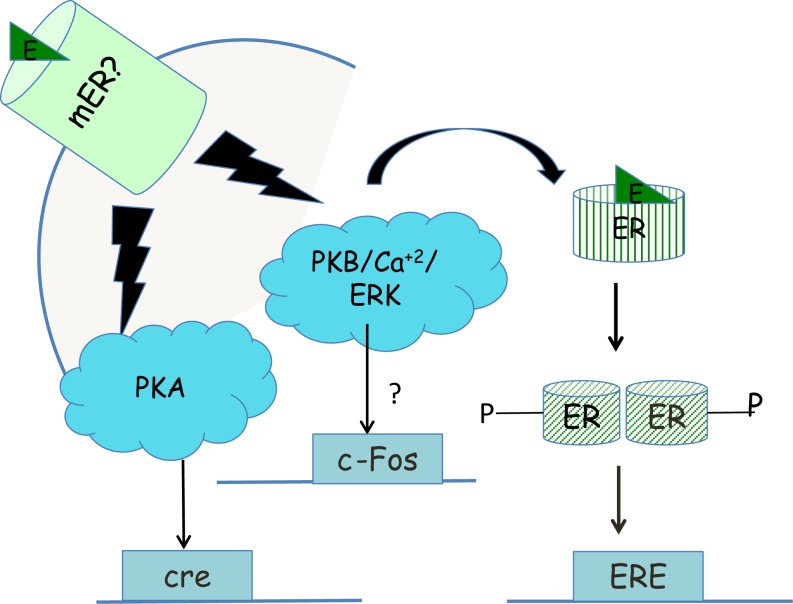
**Priming of transcription within neurons by non-genomic signaling**. Non-genomic signaling, initiated at the plasma membrane by an unknown mER can activate ERK signaling, which in turn may cause phosphorylation of the ERα and increased transcription from promoters than contain EREs (130). Alternatively, ERK signaling may also lead via unknown mechanisms to increased transcription from the c-Fos promoter (190) while PKA signaling generated by the mER may lead to increased transcription from promoters that have a cAMP response element (CRE) enhancer (189).

**Figure 4:**
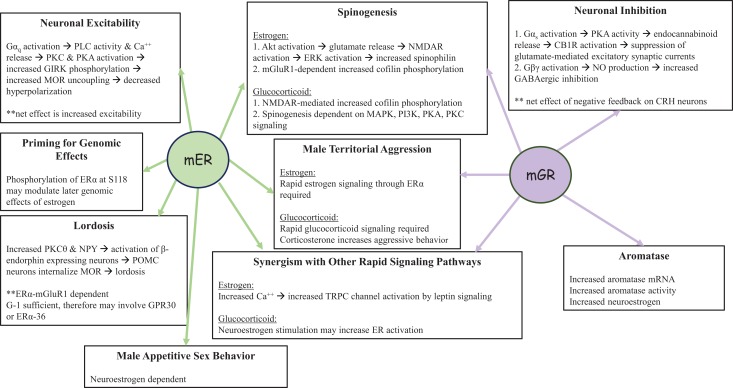
**Functions of non-genomic signaling initiated by the mER or the mGR in the CNS**. Both mER and mGR contribute to the regulation of male territorial aggression and spinogenesis in neurons, as well as synergizing with other rapid signaling pathways. In the pre-opiomelanocortin (POMC) neuron shown on the left, non-genomic signaling by the mER increases neuronal excitability while rapid negative feedback by the mGR in corticotropin releasing hormone (CRH) neurons decreases neuronal excitability (shown on the right). In addition, the mER plays a role in male sexual behavior and may interact with GPR30 regulates lordosis behavior in female rodents. Glucocorticoids via the mGR also increase neuroestrogen concentrations, via aromatase activity and the increase in aromatase protein, thus providing a route by which the actions of estrogens and glucocorticoids can converge in the brain.

One function of non-genomic signaling within a cell could be to provide cells with another pathway by which they can respond differently to the same endogenous ligand. For example, acute signaling by glucocorticoids increases aggression in an ethologically relevant context but chronic signaling by glucocorticoids increases submission (186). A parallel is seen with the regulation of the CRH neuron by acute or chronic glucocorticoid exposure via the regulation of synaptic inputs in the opposite direction. In the acute phase, rapid signaling by glucocorticoids increases negative feedback on the CRH neuron decreasing the firing of the neuron (187), while chronic exposure to glucocorticoids decreases negative feedback on the CRH and increases CRH mRNA, increasing HPA axis reactivity (188). A second function of membrane-initiated signaling might be to prime nuclear transcription via the phosphorylation of ERα, seen in a neuroblastoma cell line (130) (Figure 3), leading to greater transcription from genes with EREs in their promoters. In some cases, rapid signaling could also activate genes that do not have classical EREs in their promoters. For example, PKA signaling induced by 17β-E in the SK-N-SH neuronal cell line was required to induce the neurotensin/neuromedin gene (189), while E2-BSA in this cell line activated a reporter gene driven by a c-Fos promoter in an ERK-dependent manner (190). The idea that non-genomic signaling might prime later outputs such as behavior is also evident from the studies on sex behavior in female and male rodents. Apart from the potentiation of transcription by rapid signaling, non-genomic signaling could also decrease transcription if the decrease in transcription is ultimately important in leading to an optimal cellular response. For example, the increase in PI3K and MAPK activation that occurs within 30 min via ERα in the mHypoE-38 hypothalamic cell line is required for the long-term repression of neuropeptide Y by 17β-E (191). Third, non-genomic signaling via one pathway could also synergize with rapid signaling pathways initiated by other ligands. For instance, 17β-E-induced increases in calcium in POMC neurons augmented the activation of the calcium-sensitive TRPC channels (192) already upregulated by leptin (65). Additionally, 17β-E also transcriptionally induced PI3K p85 subunit in the hypothalamus (193, 194) and this combined with the non-genomic signaling-mediated increase in calcium potentiates the PI3K and calcium-mediated TRPC channel opening activated by leptin (70). In this case, 17β-E utilized both non-genomic, i.e., calcium increase and genomic signaling, i.e., PI3K transcriptional upregulation to synergize with the rapid signaling initiated by the leptin receptor to converge onto neuronal depolarization. Fourth, non-genomic signaling pathways may also synergize with a parallel, independent transcriptional pathway, in response to the same ligand. The rapid action of glucocorticoids in decreasing glutamatergic inputs to the CRH neuron combined with slower transcriptional downregulation of the CRH and vasopressin gene are dual pathways that achieve negative feedback regulation in the PVN (19). Glucocorticoid-mediated rapid regulation of aromatase combined with the slower transcriptional increase or aromatase mRNA and protein is yet another example where both modes would finally increase neuroestrogens in the hypothalamus. Lastly, non-genomic signaling in one cell type may also synergize with genomic signaling in another cell type to converge onto behavioral outputs. This is demonstrated by the ability of 17β-E to elicit a rapid calcium increase in hypothalamic astrocytes that is required for the increased synthesis of neuroprogesterone (195). This release of progesterone that binds to the PR that is transcriptionally induced by the liganded ERα in hypothalamic neurons is required for lordosis behavior in rodents (195). Some of these functions of non-genomic signaling are shown in Figure 4.

## Future Directions

Understanding the many facets of non-genomic signaling would also spotlight the role and source of the ligand. The idea that local estrogen synthesis in the brain, i.e., neuroestrogens is important in transducing environmental stimuli to behavior is fairly recent and has mostly been explored in birds (155, 156, 196). In addition, this would bring into focus the role of estrogens in males; traditionally, classical female-typical behaviors such as lordosis have received far more attention in the estrogen field. Second, many of the studies described above were done in rats whereas there are hints that there may be subtle differences in the mouse. For example, though there is very little GPR30 protein reported in the rat amygdala (76), there is high expression of GPR30 in the mouse basolateral amygdala and intra-amygdalar injection of G-1 attenuates behaviors that denote anxiety in the mouse (197). Similarly, though G-1 failed to regulate sex behavior in the female rat (198), it could do so in the female mouse (127). Though studies on rapid non-genomic signaling have become more mainstream, a number of mechanistic aspects of non-genomic signaling and coupled signaling remain unknown including the temporal nature of the shift from non-genomic signaling to genomic signaling. The role of the ERα variants and GR variants in any of these hypothalamically driven behaviors or in spinogenesis is unclear, with almost no studies on the subject. In this regard, it is worth nothing that studies using agonists or antagonists should be careful to verify if the effects are specific – for example, some of the G-1 mediated effects could be via ERα-36 and some of the ICI-mediated effects could be via GPR30 (Table 2).

**Table 2:** **Receptors that are activated or inhibited by the natural estrogen 17β-estradiol, the natural glucocorticoid, corticosterone, a membrane-limited estrogen conjugate (E2-BSA) or glucocorticoid-conjugate (Dex-BSA), or selective agonists or antagonists**.

Hormone/drug	Receptor activation	Receptor antagonism	Reference
17β-estradiol	All ERs	None	
E2-BSA	All mERs	None	
Propyl pyrazole triol (PPT)	ERα; 410-fold selectivity over ERβ. Also binds GPR30	None reported	(199)
R,R(THC)	ERα	ERβ	(200)
Diarylpropionitrile (DPN)	ERβ; 70-fold selectivity over ERα	None reported	(201)
ICI 182,780	GPR30	ERα/ERβ	(202)
G-1	GPR30 and ERα-36	None reported	(60)
G-15	ERα or ERβ; activates ERE	GPR30	(203)
G-36	None reported	GPR30	(203)
Corticosterone	All GRs	None reported	(204)
	Mineralocorticoid receptor	
Dexamethasone	All GRs	None reported	(204)
	MR (30% of affinity to GR)	
Dex-BSA	All mGRs	None reported	
RU-486 (mifepristone)	None reported	GR, progesterone receptor	(205)

*As can be seen, several selective agonists or antagonists show cross-reactivity to other receptors for estrogens and to other receptors for mineralocorticoids and progestins, in the case of the glucorticoids. Hence, results obtained using these compounds must be carefully interpreted*.

*MR, mineralocorticoid receptor*.

Complementary studies using deletions of the genes in animals or preferably using site-specific or temporally specific deletion would prove useful to establish specificity. The detailed molecular pathways by which 17β-E regulates spinogenesis or behaviors in the VMH are also not well worked out, though there has been considerable progress in the last decade on the receptors that might be involved. Lastly, understanding non-genomic signaling is possibly a mechanism to convey information about external physiologically relevant stimuli rapidly to the animal to generate both acute and chronic outcomes.

## Conflict of Interest Statement

The authors declare that the research was conducted in the absence of any commercial or financial relationships that could be construed as a potential conflict of interest.
